# Neglected aspects of tick-borne rickettsioses

**DOI:** 10.1186/s13071-018-2856-y

**Published:** 2018-04-24

**Authors:** Laura Tomassone, Aránzazu Portillo, Markéta Nováková, Rita de Sousa, José Antonio Oteo

**Affiliations:** 1 Department of Veterinary Sciences, University of Turin, Largo Braccini 2, 10095 Grugliasco (Torino), Italy; 2 Center of Rickettsiosis and Arthropod-Borne Diseases, Hospital San Pedro-CIBIR, C/ Piqueras 98, 26006 Logroño, Spain; 3 Department of Biology, Faculty of Medicine, Masaryk University, Kamenice 5, 625 00 Brno, Czech Republic; 4 Department of Biology and Wildlife Diseases, Faculty of Veterinary Hygiene and Ecology, University of Veterinary and Pharmaceutical Sciences Brno, Palackého 1946/1, 612 42 Brno, Czech Republic; 5 National Institute of Health Dr. Ricardo Jorge, Av. da Liberdade 5, 2965-575 Aguas de Moura, Portugal

**Keywords:** *Rickettsia* spp., Ticks, Vertebrate hosts, *Candidatus*, Epidemiology

## Abstract

Rickettsioses are among the oldest known infectious diseases. In spite of this, and of the extensive research carried out, many aspects of the biology and epidemiology of tick-borne rickettsiae are far from being completely understood. Their association with arthropod vectors, the importance of vertebrates as reservoirs, the rarity of clinical signs in animals, or the interactions of pathogenic species with rickettsial endosymbionts and with the host intracellular environment, are only some examples. Moreover, new rickettsiae are continuously being discovered. In this review, we focus on the ‘neglected’ aspects of tick-borne rickettsioses and on the gaps in knowledge, which could help to explain why these infections are still emerging and re-emerging threats worldwide.

## Background

Rickettsioses, infections caused by bacteria from the family *Rickettsiaceae*, are among the oldest known infectious diseases and are mainly transmitted by arthropod vectors [1]. The most clinically severe tick-borne rickettsiosis [2], the Rocky Mountain spotted fever, was firstly described in 1899, and ten years later Ricketts showed that the Rocky Mountain wood tick, *Dermacentor andersoni*, was vector of the causative agent of the disease [3, 4]. For approximately nine decades, *Rickettsia rickettsii* had been the only tick-borne rickettsia associated with human disease in the New World and several rickettsiae from this region were considered non-pathogenic. Similar patterns, with one known pathogenic rickettsial species and various species of unknown pathogenicity, had been observed in Europe and Africa (*Rickettsia conorii conorii*), Asia (*Rickettsia sibirica*) and Australia (*Rickettsia australis*) [2, 5].

In the past 30 years, with advent of molecular techniques, the range of known species within this group of bacteria raised significantly. Numerous rickettsial species continue to be described in a wide range of invertebrates [6–8], and generate new questions on their biology, ecology, epidemiology, geographical distribution and potential pathogenicity.

In this paper, we revised the neglected aspects of the biology of tick-transmitted *Rickettsia* spp., their relationship with tick vectors and vertebrate hosts, and the challenges of rickettsial research in a changing environment.

## Natural foci of rickettsiae and tick vectors

The genus *Rickettsia* comprises intracellular endosymbionts with remarkably adaptive potential. It is approximately 150 million years old, and splits into two main clades, one primarily infecting arthropods, and the second infecting a variety of other eukaryotes, such as protists and leeches [7]. Around 24% of terrestrial arthropod species are thought to be infected with *Rickettsia* endosymbionts [8].

The best-known members of this bacterial genus are human pathogens associated with blood-feeding arthropods. Humans are accidental hosts, except for *Rickettsia prowazekii*, for which they are reservoir [9]. Reconstructed phylogeny of the genus *Rickettsia*, based on whole genome sequence data, showed that hard ticks (Ixodidae) are found across phylogeny, which correlates with the fact that they are ancestral arthropod hosts for rickettsiae [10]. Most tick-borne rickettsiae belong to the spotted fever group (SFG). They are maintained in nature by transstadial and transovarial transmission in ticks [11], so it is generally stated that ticks act both as vector and as reservoir of most SFG species [12]. *Rickettsia*-free ticks may acquire the bacteria by feeding on rickettsemic host, co-feeding or sexual transmission [13–15].

Looking at the associations with tick genera [1, 2], it is evident that the level of host specificity varies among rickettsial species. Some of them seem to be strictly linked with one tick vector, such as *R. conorii* with *Rhipicephalus sanguineus* (*s.l.*) ticks (with the exception of *R. conorii caspia* transmitted by *Rh. pumilio* in the Caspian Sea region), and *Rickettsia* sp. 364D with *Dermacentor occidentalis* [2]. Other rickettsiae are related to tick species belonging to the same genus, such as *Rickettsia slovaca*, which infects the two *Dermacentor* species present in Europe, *D. marginatus* and *D. reticulatus* [11, 16]. Finally, *R. rickettsii* is an example of a *Rickettsia* associated with a broad spectrum of tick species belonging to different genera [2].

Most *Rickettsia* species are transmitted by hard ticks, but rickettsiae in soft ticks (Argasidae) are continuously being identified (Table 1). Even though there are no reports of human cases due to soft-ticks bites so far, the ability of argasid ticks to transmit rickettsiae to vertebrates and the possible implications to human and animal health are worth further study. In fact, among the identified rickettsiae, some are pathogenic (i.e. *R. felis*). Moreover, among the soft tick species infected by rickettsiae, some commonly feed on humans, such as *Carios capensis*, *Ornithodoros erraticus* and *O. moubata*; in particular, the latter two tick species are already recognized vectors of tick-borne relapsing fever borreliae to humans [17].

**Table 1:** *Rickettsia* species associated with soft ticks

*Rickettsia* species^a^	Methodology (detected/isolated)	Tick species	Animal or site association	Geographical origin	Reference
*R. bellii*	Detected	*Carios capensis*	Seabirds	Western Indian Ocean Islands	[91]
*R. felis*	Detected	*Carios capensis*	Brown pelican nests	USA	[92]
*R. hoogstraalii*	Detected	*Argas persicus*	Human dwellings, trees	Ethiopia	[93]
	Detected	*Carios capensis*	Seabirds	Japan	[94]
	Isolated	*Carios capensis*	ns	USA	[95]
	Detected	*Carios capensis*	Seabirds	Western Indian Ocean Islands	[91]
	Detected	*Carios sawaii*	Seabirds	Japan	[94]
*R. lusitaniae*	Isolated	*Ornithodoros erraticus*	Pigs/pigpens	Portugal	[96]
	Detected	*Ornithodoros yumatensis*	Bat caves	Mexico	[97]
*R. nicoyana*	Isolated	*Ornithodoros knoxjonesi*	*Balantiopteryx plicata*	Costa Rica	[98]
*R. wissemanii*	Detected	*Ornithodoros hasei*	Bats	French Guiana	[99]
*R. argasii*	Isolated	*Argas dewae*	Bats boxes	Australia	[2]
*Rickettsia* spp.	Isolated	*Argas persicus*	ns	Armenia	[100]
	Detected	*Argas vespertilionis*	Bat-infested building	France	[101]
	Detected	*Carios capensis*	Yellow-legged gull nests	Algeria	[102]
	Detected	*Carios kelleyi*	Bat-infested building	USA	[103]
	Detected	*Ornithodoros erraticus*	Rodent burrows	Algeria	[102]
	Detected	*Ornithodoros moubata*	Human dwellings	Tanzania	[104]
	Detected	*Ornithodoros rupestris*	Rodent burrows	Algeria	[102]

^a^Includes *Rickettsia*-like species

*Abbreviation*: ns, not specified

Rickettsiae can have deleterious effects on their invertebrate hosts; for example, they have been shown to determine parthenogenesis in Hymenoptera, male killing in Coleoptera or larger body size in leeches (Hirudinida) [6, 18]. For ixodid ticks, negative effects can occur on their viability and survival, on the oviposition and percentage of successful transovarial transmission within each arthropod generation. These effects have been observed in ticks infected in the laboratory with different rickettsiae [11, 19–22], and are more evident when pathogenic species [2] infect ticks (e.g. *R. rickettsii* and *R. conorii*), compared to less (e.g. *Rickettsia* sp. strain Atlantic rainforest) or non-pathogenic species (e.g. *R. montanensis*, *R. bellii* and *R. rhipicephalii*). Interestingly, external factors, such as temperature, can influence these phenomena. For instance, *Rh. sanguineus* (*s.l.*) can maintain *R. conorii conorii* for several generations, but naturally infected specimens showed higher mortality, compared to uninfected ticks, when exposed to low temperatures (4 °C), so that winter colds may explain the low prevalence of *R. conorii*-positive ticks in nature [23]. Conversely, *D. andersoni* ticks survived better to experimental *R. rickettsii* infection when incubated at 4 °C [19]. The loss of performance in ticks induced by rickettsial infections, and interactions with environmental factors, are still poorly understood.

Recent studies have also addressed the positive influence of rickettsial endosymbionts on tick hosts. For example, metabolic reconstructions of rickettsial genomes in *Ixodes scapularis* and *I. pacificus* ticks showed that all genes required for folic acid biosynthesis are present in rickettsial genomes [24]. Later, it was proven that *Rickettsia* species phylotype GO21 of *I. pacificus* produces an enzyme involved in the vitamin B9 biosynthesis, which indicates nutritional interactions between this endosymbiont and its host [25].

Other studies revealed that rickettsial endosymbionts have negative effects on pathogenic rickettsiae within the tick vector and preclude their secondary infection, through a rickettsial interference in ovarian tissue colonization, or transovarial transmission [19, 26, 27]. For instance, this interference was observed between *R. peacockii* and *R. rickettsii* [28], *R. rhipicephali* and *R. montana* [26] and, more recently, between rickettsial species in different families, i.e. between *R. bellii* and *A. marginale* in *D. andersoni* ticks [29]. Therefore, *Rickettsia* endosymbionts are thought to protect ticks against tick-borne pathogens colonization, and to condition their abundance and diversity [30]. However, positive interactions with pathogens can also occur, as was shown between *Rickettsia* spp. and *Borrelia lusitaniae* in lizards’ ticks [31]. The simultaneous occurrence of multiple pathogens in ticks is a topic of concern, since their concurrent transmission to vertebrate hosts can have severe health consequences for patients [32].

The study of the interactions of endosymbionts with ticks and with pathogens within ticks, and among different pathogens, is still on its infancy but has a promising future. Moreover, it has the very fascinating perspective of using tick microbiome manipulation to limit the transmission and maintenance of pathogens in ticks, thus decreasing their vectorial competence [29, 30]. However, the unavailability of a suitable axenic medium for rickettsiae culture hampers many conventional genetic approaches. Recent identification of 51 host metabolites required by *Rickettsia*, which offered information about host-dependent metabolism, may help dissociating rickettsiae from eukaryotic cells [33].

## *Rickettsia* spp. infection in animals

As previously said, ticks are usually thought to be the main reservoir of SFG rickettsiae thanks to transstadial and transovarial transmission. However, studies have demonstrated the occurrence of transovarial transmission in only a limited number of species [11]. Moreover, the efficiency of vertical transmission varies according to the rickettsial and tick species [19, 34], and transmission may not occur in female ticks infected by sexual/co-feeding transmission [35]. Vertebrate hosts may thus be necessary to maintain and perpetuate some rickettsial agents in nature [36]. However, systemic transmission of infection from the vertebrate host to feeding ticks seems to have a limited importance, because rickettsemia in most vertebrates occurs at low level and/or it is transient [37]. In fact, few studies provide evidence that animal species are competent reservoirs of *Rickettsia* spp. Generally, we consider as ‘reservoir’ a host that permanently maintains a pathogen and is able to transmit it to the target population/vector [38]. To determine potential reservoirs, studies identifying natural infection in hosts (through antibody detection, isolation of the infectious agent, or its genes from the host) may be useful, together with xenodiagnostic experiments, to show the host susceptibility to the infection and its ability to transmit the pathogen to the vector. In the case of *Rickettsia* spp., natural infection has been demonstrated in a number of vertebrate species, but the studies rarely investigated the persistence of the agent in the host. In regards to xenodiagnostic experiments, most of them have shown that only a small percentage of uninfected feeding ticks were able to acquire the infection from the rickettsemic host (Table 2).

**Table 2:** Natural and experimental infections by tick-borne *Rickettsia* spp. in vertebrate host species

*Rickettsia* species	Host species	Type of infection	Methodology demonstrating the infection	Reference
*R. rickettsii*	Birds (Ciconiiformes, Piciformes, Passeriformes)	Natural	Antibodies	[41]
	Capybara (*Hydrochoerus hydrochaeris*)	Experimental	Rickettsemia, infection transmitted to *Amblyomma cajennense* ticks (25–30% success)	[44]
	Rodents and lagomorphs	Natural	Isolation	[41, 105]
		Experimental	Rickettsemia, infection transmitted to *Dermacentor andersoni* ticks	[40, 42]
	Opossum (*Didelphis* spp.)	Natural	Isolation	[41, 106, 107]
		Experimental	Rickettsemia, infection transmitted to *Amblyomma cajennense* ticks (5% success)	[41, 43]
	White-tailed deer (*Odocoileus virginianus*)	Natural	Antibodies	[41]
	Wild carnivores (fox, raccoon, skunk)	Natural	Antibodies	[41]
	Dog (*Canis lupus familiaris*)	Experimental	Antibodies	[52^a^]
		Natural	Antibodies, DNA in blood	[53^a^, 59, 60]
	Horse (*Equus caballus*)	Experimental	Antibodies	[108]
*R. parkeri*	Opossum (*Didelphis* spp.)	Experimental	DNA in blood	[109]
	Dog (*Canis lupus familiaris*)	Natural	Antibodies, DNA in blood	[110, 111]
*R. conorii*	Lagomorphs (*Oryctolagus cuniculus*, *Lepus granatensis*)	Natural	Antibodies	[112, 113]
	Cat (*Felis catus*)	Natural	Antibodies	[114]
	Dog (*Canis lupus familiaris*)	Experimental	Antibodies, infection transmitted to *Rh. sanguineus* ticks	[39]
		Natural	Antibodies, DNA in blood	[54–56^a^, 58]
*R. conorii*-like	Bats (African species)	Natural	DNA in blood	[115]
*R. helvetica*	Deer (*Cervus nippon*, *Capreolus capreolus*)	Natural	DNA in blood	[116, 117]
	Passerine birds (*Erithacus rubecula*, *Parus major*, *Prunella modularis*)	Experimental (in *Parus major*)	Infection transmitted to *Ixodes ricinus* ticks	[46]
		Natural	DNA in blood	[118]
	Hedgehog (*Erinaceus europaeus*)	Natural	DNA in tissues	[119]
	Lizard (*Teira dugesii*, *Podarcis muralis*)	Natural	DNA in tissues	[120, 121]
	Small rodents (*Myodes glareolus*, *Microtus arvalis*, *M. arvestis*, *Apodemus flavicollis*, *A. sylvaticus*, *Mus musculus*)	Natural	Antibodies, DNA in tissues	[117, 122–124]
	Wild boar (*Sus scrofa*)	Natural	DNA in blood	[117]
	Dog (*Canis lupus familiaris*)	Natural	Antibodies	[61]
*R. massiliae*	Cat (*Felis catus*)	Natural	Antibodies	[114]
*R. monacensis*	Lizard (*Teira dugesii*)	Natural	DNA in tissues	[120]
	Dog (*Canis lupus familiaris*)	Natural	DNA in blood	[125]
*R. raoultii*	Dog (*Canis lupus familiaris*)	Natural	Antibodies, DNA in blood	[61, 126]
*R. slovaca*	Small rodents (*Apodemus* spp., *Myodes glareolus*)	Natural	DNA in tissues	[50]
		Experimental	Antibodies	[127]
	Wild boar (*Sus scrofa*)	Natural	DNA in tissues	[128–130]
	Cattle (*Bos taurus*)	Natural	Antibodies	[131]
	Dog (*Canis lupus familiaris*)	Natural	Antibodies	[61]
	Goat (*Capra hircus*)	Natural	Antibodies	[131]
		Experimental (American strain)	DNA in tissues, infection transmitted to *Dermacentor variabilis* ticks (< 5% success)	[45^a^]
	Sheep (*Ovis aries*)	Natural	Antibodies	[131]

^a^Studies reporting clinical signs in animals

The dog was identified as reservoir of *R. conorii* by means of experimental studies. In fact, dogs infected both by inoculation and by using infected ticks were able to infect feeding *Rh. sanguineus* (*s.l.*) larvae and nymphs [39]. In turn, these ticks were able to transmit the rickettsiae to the next developmental stages and to naïve dogs. Dogs maintained the ability to transmit the infection to ticks for at least one month post-infection, also when rickettsemia was not detectable by PCR. They developed antibodies within two weeks after the infection, which declined within three to six months, and their persistence and titres were shown to be dose dependent [39].

Rickettsial transmission to feeding ticks was also demonstrated, in the Americas, in opossums, lagomorphs, capybaras, small rodents (*R. rickettsii*) [40–44] and goats (North American isolate of *R. slovaca*) [45]. In Europe, Heylen et al. [46] showed that experimentally infected great tits (*Parus major*) transmit *R. helvetica* to feeding *I. ricinus* and facilitate co-feeding.

Co-feeding is in fact a transmission mechanism, which may be very important in perpetuating rickettsiae in nature [20, 47]. It can occur among both infected and uninfected ticks simultaneously feeding in close proximity on the host’s skin, and by 'extended co-feeding transmission' in a localized site, after the infected ticks have dropped off [48]. Local skin infection may facilitate the transmission, thanks to the tropism of SFG rickettsiae for the endothelial cells of peripheral blood vessels [49]. As an example, natural transmission of *R. conorii israelensis* was shown among *Rh. sanguineus* (*s.l.*) ticks co-feeding on dogs. The transmission was less efficient when co-feeding occurred on seropositive dogs [47], but Levin et al. [49] observed that it might remain efficient at high densities of co-feeding ticks. Therefore, high tick aggregation levels on the same individuals can favour *Rickettsia* maintenance in the tick populations. Recent studies suggest the occurrence in nature of this non-systemic transmission, in animals that are preferred hosts for the immature stages of tick vectors (e.g. small mammals, passerine birds), especially in heavily infested individuals [46, 50]. In this sense, vertebrates act as ‘amplifiers’ of rickettsiae, contributing to its spread in the ecosystem, even in the absence of a systemic infection. However, Paddock et al. [51] recently suggested that ticks could acquire SFG rickettsiae from ear tissues of systemically infected hosts; in fact, they observed rickettsial aggregates, persisting for at least 14 days, in the ear dermis of guinea pigs inoculated intraperitoneally with *Rickettsia* sp. Black Gap. Therefore, we need further research to clarify the relative importance of systemic and non-systemic transmission mechanisms for the maintenance of *Rickettsia* spp.

In general, clinical illness is not reported in animals infected by tick-borne rickettsiae. Cases of illness have occurred in dogs infected by *R. rickettsii* [52, 53] and *R. conorii* [54–56] in endemic areas. Clinical signs were similar to humans’, including fever, lethargy, anorexia, depression, cutaneous petechiae and ecchymoses, epistaxis, conjunctivitis, ocular discharge, lymph node enlargement, diarrhoea, weight loss and dehydration. Haematological abnormalities included anaemia, thrombocytopenia and leucocytosis. In the case of *R. conorii*, symptoms are generally mild and of short duration, so that the disease goes unnoticed [39]. In any case, the clinical diagnosis in dogs is challenging, since other pathogens can cause similar clinical signs, such as *Ehrlichia canis*, *Anaplasma platys*, *Babesia canis* and *Hepatozoon canis* [55]. Studies from distinct geographical regions showed the presence of antibodies and DNA of other *Rickettsia* species in dogs’ blood (Table 2). Some of these rickettsiae are pathogenic to humans (e.g. *R. helvetica*, *R. slovaca*, *R. parkeri* and *R. raoultii*), but all observed animals were asymptomatic.

Actually, the mechanisms determining the different pathogenicity and virulence of rickettsial species are poorly understood, even in humans [57]. Future research on the interactions between SFG rickettsiae and the host intracellular environment could help in understanding why most animals do not show any clinical sign when infected by rickettsiae that are pathogenic to humans. In addition, histologic studies in mammals would be valuable, in order to characterize specific features related to *Rickettsia* spp. infections (e.g. changes in tissues due to vasculitis).

Finally, it is important to remember that animals serve as sentinels for rickettsial circulation and are useful for rickettsiosis surveillance in humans. For example, natural antibodies to SFG rickettsiae were detected in dogs living in close proximity to human cases of Mediterranean spotted fever [58], and Rocky Mountain spotted fever [59, 60]. Dogs seropositive to *R. helvetica*, *R. raoultii* and *R. slovaca* were reported in Austria [61], indicating a contact with SFG species prevalent in continental Europe. Generally, high prevalence of rickettsial infection in ticks parasitizing dogs correlates with high levels of SFG rickettsiae antibodies in dogs [55, 56, 62, 63].

## New Rickettsiae

During the last decades, molecular techniques have allowed researchers to genetically characterize several bacteria within the genus *Rickettsia* before they have been cultured. According to taxonomic criteria proposed by experts, these uncultured rickettsiae can be given *Candidatus* (*Ca*.) status and are considered potential new species of *Rickettsia* [64].

In regards to *Rickettsia* culture, ‘temperature’ and ‘enrichment of medium’ variables seem to be essential to establish a pure culture. Different cell lines, media with supplementations and culture conditions have been tested for the *in vitro* growth of *Rickettsia* spp. [65]. Nevertheless, the isolation of these bacteria is still a challenge that not always succeeds. Thus, several genotypes of *Rickettsia* were observed in DEBONEL/TIBOLA patients, which were different from validated *Rickettsia* species involved as human pathogens [66]. One of them is *Rickettsia rioja*, which has been molecularly characterised and detected from human blood and biopsies, and ticks, but to date remains uncultured [67, 68].

The number of microorganisms that fulfil criteria of “*Ca.* Rickettsia” spp. continues to increase in Europe and in other continents (Tables 3 and 4). If the analyses of additional rickettsial genes are needed to define the taxonomic status of a novel rickettsial organism, it must be designated as a strain. This was, for instance, the case of *Rickettsia* sp. strain Davousti. It was first detected in *Amblyomma tholloni* from African elephants in 2007 [69], and further genetically characterized as “*Ca.* Rickettsia davousti” from one *Amblyomma* nymph attached to a traveller from Gabon to Spain in 2015 [70]. Table 5 details the new strains of *Rickettsia*.

**Table 3:** “*Candidatus* Rickettsia” spp. in Europe

“*Ca.* Rickettsia” spp.	Associated arthropod or source	Country of the first identification	Reference	Associated human disease	Reference of associated disease
“*Ca.* R. tarasevichiae”	*Ixodes persulcatus*	Russia	[132]	“*Ca.* R.tarasevichiae” infection	[133]
“*Ca.* R. kotlanii”	*Ixodes* spp.	Hungary	[134]	–	–
“*Ca.* R. barbariae”	*Rhipicephalus turanicus*	Italy	[135]	–	–
“*Ca.* R. rioja”	*Dermacentor marginatus*	Spain	[67]	DEBONEL/TIBOLA	[68]
“*Ca.* R. siciliensis”	*Rhipicephalus turanicus*	Italy	[136]	–	–
“*Ca*. R. uralica”	*Ixodes trianguliceps*	Russia	[137]	–	–
“*Ca*. R. mendelii”	*Ixodes ricinus*	Czech Republic	[138]	–	–

**Table 4:** “Candidatus Rickettsia” spp. outside Europe

“*Ca.* Rickettsia” spp.	Associated arthropod or source	Country of the first identification	Reference of the first identification	Associated human disease	Reference of associated disease
“*Ca.* R. andeanae”	*Amblyomma maculatum*, *Ixodes boliviensis*	Perú	[139]	–	–
“*Ca.* R. kellyi”	Unknown arthropod (detected in a human skin biopsy from a maculopapular lesion)	India	[140]	Unnamed	[140]
“Ca. R. principis”	*Haemaphysalis japonica*	Russia	[141]	–	–
“*Ca.* R. tasmanensis”	*Ixodes tasmani*	Australia	[142]	–	–
“*Ca.* Rickettsia” sp. strain Argentina	*Amblyomma parvum*, *Amblyomma pseudoconcolor*	Argentina	[143]	–	–
“*Ca.* R. cooleyi”	*Ixodes scapularis*	USA	[144]	–	–
“*Ca*. R. hebeiii”	*Haemaphysalis longicornis*	China	[145]	–	–
“*Ca*. R. liberiensis”	*Ixodes muniensis*	Liberia	[146]	–	–
“*Ca*. R. kulagini”	*Rhipicephalus annulatus*	Kenya	[147]	–	–
“*Ca*. R. angustus”	*Ixodes angustus*	Canada	[148]	–	–
“*Ca.* R. kingi”	*Ixodes kingi*	Canada	[149]	–	–
“*Ca.* R. senegalensis”	*Ctenocephalides felis* (cat flea)	Senegal	[150]	–	–
“*Ca.* R. davousti”	*Amblyomma* sp. (attached to a human)	Gabon	[70]	–	–
“*Ca.* R. sepangensis”	*Amblyomma varanense*	Malaysia	[151]	–	–
“*Ca*. R. johorensis”	*Amblyomma helvolum*, *Amblyomma varanense*	Malaysia	[151]	–	–
“*Ca*. R. goldwasserii”	*Haemaphysalis* spp., *Rhipicephalus* spp.	Palestine	[152]	–	–
“*Ca*. R. gannanii”	*Haemaphysalis qinghaiensis*	China	[153]	–	–
“*Ca. R.* indica”	Human blood	Imported from India to Japan (traveler)	[154]	Unnamed	[154]
“*Ca*. R. moyalensis”	*Rhipicephalus appendiculatus*	Kenya	[155]	–	–
“*Ca*. R. wissemanii”	*Ornithodoros hasei* (soft tick)	French Guiana	[100]	–	–

**Table 5:** Strains of *Rickettsia* spp. (without *Candidatus* status)

*Rickettsia* sp. strain	Associated arthropod or source	Country of the first identification	Reference of the first identification	Associated human disease	Reference of associated disease
*Rickettsia* sp. strain Uilenbergi	*Amblyomma tholloni*	Central African Republic	[69]	–	–
*Rickettsia* sp. Atlantic rain forest	*Amblyomma ovale* *Rhipicephalus sanguineus*	Brazil	[156]	Unnamed	[157]
*Rickettsia* sp. strain Pampulha	*Amblyomma dubitatum*	Brazil	[158]	–	–
*Rickettsia* sp. strain colombianensi	*Amblyomma dissimile*	Colombia	[159]	–	–
*Rickettsia* sp.- novel isolate	*Ixodes ricinus*	Czech Republic	[160]	–	–
*Rickettsia* sp. strain Tselenti	*Hyalomma anatolicum excavatum* *Rhipicephalus turanicus*	Cyprus	[161]	–	–
*Rickettsia* sp. strain IbR/CRC	*Ixodes boliviensis*	Costa Rica	[162]	–	–

The association of a bacterium with human disease can be found even 65 years after its discovery, as happened with *Rickettsia parkeri* [71]. Therefore, all *Rickettsia* organisms (species, *Ca.* or strains) must be considered as potential human pathogens.

In a near future, exploring the bacteriome of arthropods (ticks, fleas, mosquitoes and others) will mean a step forward for the knowledge of potential new *Rickettsia* species and for the improvement of diagnosis and treatment of vector-borne diseases [72].

## Rickettsioses in a changing environment

Due to the close relationship between ticks and vectors, changes in the distribution of arthropods are very important for the epidemiology of rickettsiosis. Climate, density of vertebrate hosts, landscape features and anthropogenic factors are the drivers of such changes [73]. While these may affect in a relative way rickettsial agents with widespread distribution of vectors (e.g. the flea-transmitted *R. typhi* and *R. felis*, or *R. prowazekii*, transmitted by the body louse), they can have greater importance for tick-borne rickettsioses, most of which are restricted to specific endemic areas. For instance, *D. reticulatus* colonization of new areas in Eastern Europe was associated to cases of DEBONEL/TIBOLA in the human population [74]. In the case of *I. ricinus*, its geographical expansion [73] has not been accompanied so far with an increase of human cases of associated rickettsioses (caused by *R. helvetica* and *R. monacensis*). This could be due to a low competence of *I. ricinus* as a vector of these *Rickettsia* species and/or to their low pathogenicity [75].

As regards climate changes, it is known that, for example, warming has an impact on the activity and aggressiveness of the brown dog tick *Rh. sanguineus* (*s.l.*), increasing human attacks and the possibility of transmission of severe rickettsioses [76].

The availability of vertebrate animals that are common tick hosts may favour the maintenance and transmission of *Rickettsia* spp. The introduction of wild animals in urban areas may facilitate interchange of arthropods with domestic animals, increasing human exposure to *Rickettsia* spp. [77]. *Vice versa*, pathogens infecting domestic animals may threaten the health of wild animals when they share the same habitat (e.g. livestock and Andean tapirs in South America) [78]. Moreover, birds are possible dispersers of *Rickettsia* spp. and other tick-borne pathogens [79, 80]. It is known that migratory birds respond to environmental changes and are able to adjust their timing of migration according to climate. This can affect the life-cycle of ticks feeding on them and, consequently, the potential transmission pattern of tick-borne pathogens.

Social changes (e.g. demographics, availability of public health care infrastructures, human behaviour, trade and travel, economic development, war and famine) may also have an impact on vector dynamics and alter pathogen adaptation or evolution. Regarding human behaviour, outdoor activities have increased in the last decades by leisure or due to the economic crisis (e.g. picking mushrooms for trade), accompanied by the risk of being bitten by ticks [81].

International trade and travel are likely routes of introduction of rickettsiosis. In fact, travel-acquired rickettsioses are frequently considered imported diseases. Several travel-associated infections refer to tourists infected by *R. africae* in sub-Saharan Africa, who develop African tick-bite fever (ATBF) [82]. It has been suspected that tick-borne infections can also affect individuals who have recently been visited by travellers, as was the case of the first DEBONEL/TIBOLA related to a *D. marginatus* bite in a patient without travel history documented in United Kingdom, where this tick species had not been notified yet [83].

## Conclusions

We live in a changing world and we will probably have to face threats to health more and more frequently. The recent finding of the tick-borne encephalitis (TBE) virus in ticks and roe deer from a forested area in the Netherlands [84] led to the identification of the two first autochthonous cases of TBE in this country [85, 86]. Similarly, the description of the two first cases of Crimean-Congo haemorrhagic fever (CCHF) in Spain in 2016 [87], followed the identification of the etiologic agent (CCHF virus) in ticks from southern Europe several years before [88]. Could we also expect the emergence of tick-borne rickettsioses? Indeed, we do not know the actual impact of rickettsial diseases in Europe. The main sources of information we have are published papers, but often only impact reports are available to the scientific community, while other relevant data are less accessible (e.g. grey literature). In addition, official information is not consistently updated; for example, the last technical report on tick-borne rickettsioses from the ‘European Centre for Disease Prevention and Control’ (ECDC) dates back to October 2013 and includes data up to 2010 [89]. In order to tackle emerging threats in a more timely and effective way, a major effort is required in Europe to harmonize data collection and notification on rickettsioses (and other tick-borne diseases). Furthermore, surveillance could be strengthened by sharing of information and inter-disciplinary collaboration among public, animal and environmental health, based on a ‘*One Health*’ approach [90].
